# Prolyl oligopeptidase inhibition reduces PolyQ aggregation and improves cell viability in cellular model of Huntington’s disease

**DOI:** 10.1111/jcmm.14675

**Published:** 2019-09-29

**Authors:** Susanna Norrbacka, Dan Lindholm, Timo T. Myöhänen

**Affiliations:** ^1^ Division of Pharmacology and Pharmacotherapy University of Helsinki Helsinki Finland; ^2^ Medicum Department of Biochemistry and Developmental Biology University of Helsinki Helsinki Finland; ^3^ Minerva Foundation Institute for Medical Research Biomedicum Helsinki 2U Helsinki Finland

**Keywords:** autophagy, huntingtin, neurodegeneration, protein aggregation, protein processing

## 1 INTRODUCTION

Unlike several other neurodegenerative diseases, Huntington's disease (HD) has clear genetic background where huntingtin gene (*HTT*) has an abnormally expanded CAG repeat near the N terminus. This causes production of mutant huntingtin (mHtt) protein with polyglutamine‐expanded (polyQ) tail, and over 36 CAG repeats cause aggregation‐prone polyQ tail. This leads to accumulation of intracellular aggregates of mHtt in the cell producing toxicity with deleterious effects on gene transcription, protein processing and cell signalling cascades ultimately leading to neuronal death (for review, see Ref.^1^). Clinically, HD is characterized by severe symptoms such as impaired movement and chorea as well as declined cognitive, and there are currently no rational treatments. Recently, great interests have been devoted to the possibility to reduce mHtt burden by lowering the expression levels of mHtt using DNA‐ or RNA‐targeted therapies. However, these approaches are in their infancy and may bear risks related to complete loss of Htt that may have toxic effects.^2^

An alternative way to interfere with the aggregates would be to increase the degradation rate of mHtt. As a background to this is the finding that mHtt aggregates are known to impair the ubiquitin‐proteasome system causing a further increase in the intracellular protein load in cells. In addition, autophagy inducers can produce beneficial effects in cell culture and animal models of HD (for review, see Ref.^3^). However, the potential toxicity and adverse effects of such autophagy inducers may also cause serious drawbacks that has to be taken into account.^4^

Prolyl oligopeptidase (PREP) is mainly cytosolic enzyme involved in peptide bond cleavage that was recently shown to negatively regulate autophagy.^5^ KYP‐2047 is a small‐molecule inhibitor of PREP having a disease‐modifying effect in the alpha‐synuclein (aSyn) models of Parkinson´s disease (for review, see Ref.^6^). We have shown that KYP‐2047 reduced the amount of aSyn‐aggregates in in vitro and in vivo by activating autophagy, while PREP directly interacts with aSyn and colocalizes with aSyn in Parkinsonian brain.^6^, ^7^ Interestingly, we observed that PREP is also expressed in the striatum and particularly in the medium spiny neurons^8^ that preferentially degenerate in HD.^1^ This prompted us to study whether the inhibition of PREP could interfere with mHtt and reduce its aggregation in cell models of HD.

## 2 MATERIALS AND METHODS

### 2.1 Reagents

Reagents were purchased from Sigma‐Aldrich if not otherwise specified. See Materials S1 for details.

### 2.2 Cell cultures

mHtt stably expressing HeLa cells having the first 17 amino acids of Htt linked to a 103Q polyQ repeat tagged to green fluorescent protein (GFP)^9^ were used in the study. See Materials S1 for details.

### 2.3 Cell viability assay

Cell viability was studied with basic cell viability assays.^10^ See Materials S1 for details.

### 2.4 Western blot

Immunoblotting was performed by using standard SDS‐PAGE and transfer methods as previously described.^5^ More detailed protocol and antibody details are presented in Materials S1.

### 2.5 Immunocytochemistry (ICC)

ICC was used to detect Htt aggregation after proteasomal inhibition as described earlier.^11^ Detailed protocol is presented in Materials S1.

### 2.6 Statistical analysis

Statistical analyses were performed using GraphPad Prism (version 6.02, GraphPad Software), and the one‐way ANOVA test with Tukey's post hoc comparison and multiple Student's t test. Data are presented as mean ± SEM, and differences were considered statistically significant at *P* < .05.

## 3 RESULTS AND DISCUSSION

### 3.1 PREP inhibition attenuates mHtt 103Q toxicity after proteasomal inhibition

**Figure 1:**
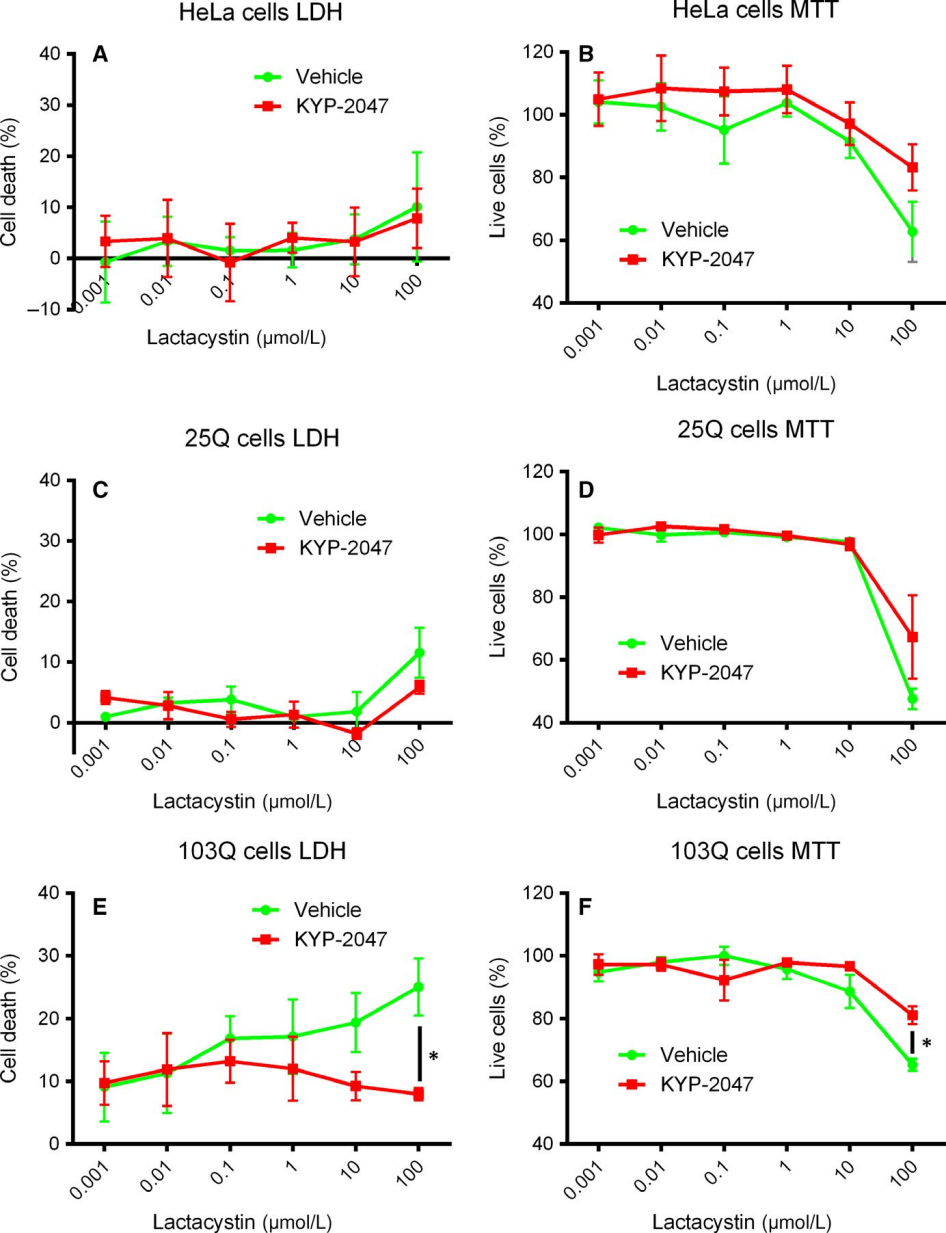
KYP‐2047 attenuates lactacystin‐induced toxicity in 103Q‐expressing HeLa cells. Wild‐type (A‐B)‐, 25Q (C‐D)‐ and 103Q (E‐F)‐expressing HeLa cells were exposed to increasing amounts of a proteasomal inhibitor, lactacystin (1 nmol/L to 100 µmol/L). About 100 µmol/L of lactacystin was particularly toxic for 103Q HeLa cells (E) in LDH assay and 1 µmol/L KYP‐2047 significantly decreased LDH release. About 100 µmol/L lactacystin was toxic for all cell lines but in 103Q HeLa, 1 µmol/L KYP‐2047 attenuated lactacystin toxicity (F).*P < .05, Student's *t* test (n of parallel samples = 3; n of individual experiments = 3)

Wild‐type HeLa cells, and HeLa cells expressing either 25Q‐ or 103Q‐containing Htt protein were cultured for 48 hours in the presence of 1 nmol/L to 100 µmol/L of lactacystin. This is to model deficits in the protein processing that is common characteristic of HD.^12^ Increasing the concentration of lactacystin reduced cell viability specifically of 103Q‐expressing cells using the LDH assay and this could be reversed using 1 µmol/L KYP‐2047 (Figure 1A,C,E; *P* < .05, multiple Student's *t* test). Interestingly, 100 µmol/L lactacystin reduced the MTT signal in wild‐type‐ and 25Q‐expressing cells, and KYP‐2047 did not attenuate this (Figure 1B,D). In 103Q cells, PREP inhibition significantly improved cell viability also in the MTT assay (Figure 1F; *P* < .05, multiple Student's *t* test).

Data show that high‐dose of lactacystin is toxic for cells even without polyQ overexpression. However, significant and dose‐dependent toxicity in LDH assay was seen only in 103Q‐expressing cells, indicating that longer polyQ tract increases toxicity similar to human HD.^1^ LDH measures cell membrane damages, and plasma and organelle membrane damage are associated with mHtt aggregation in HD.^1^ Therefore, it seemed worthwhile to study whether addition of KYP‐2047 would affect the aggregation of mHtt protein in these cells.

### 3.2 PREP inhibition reduces mHtt 103Q aggregates in cells

**Figure 2:**
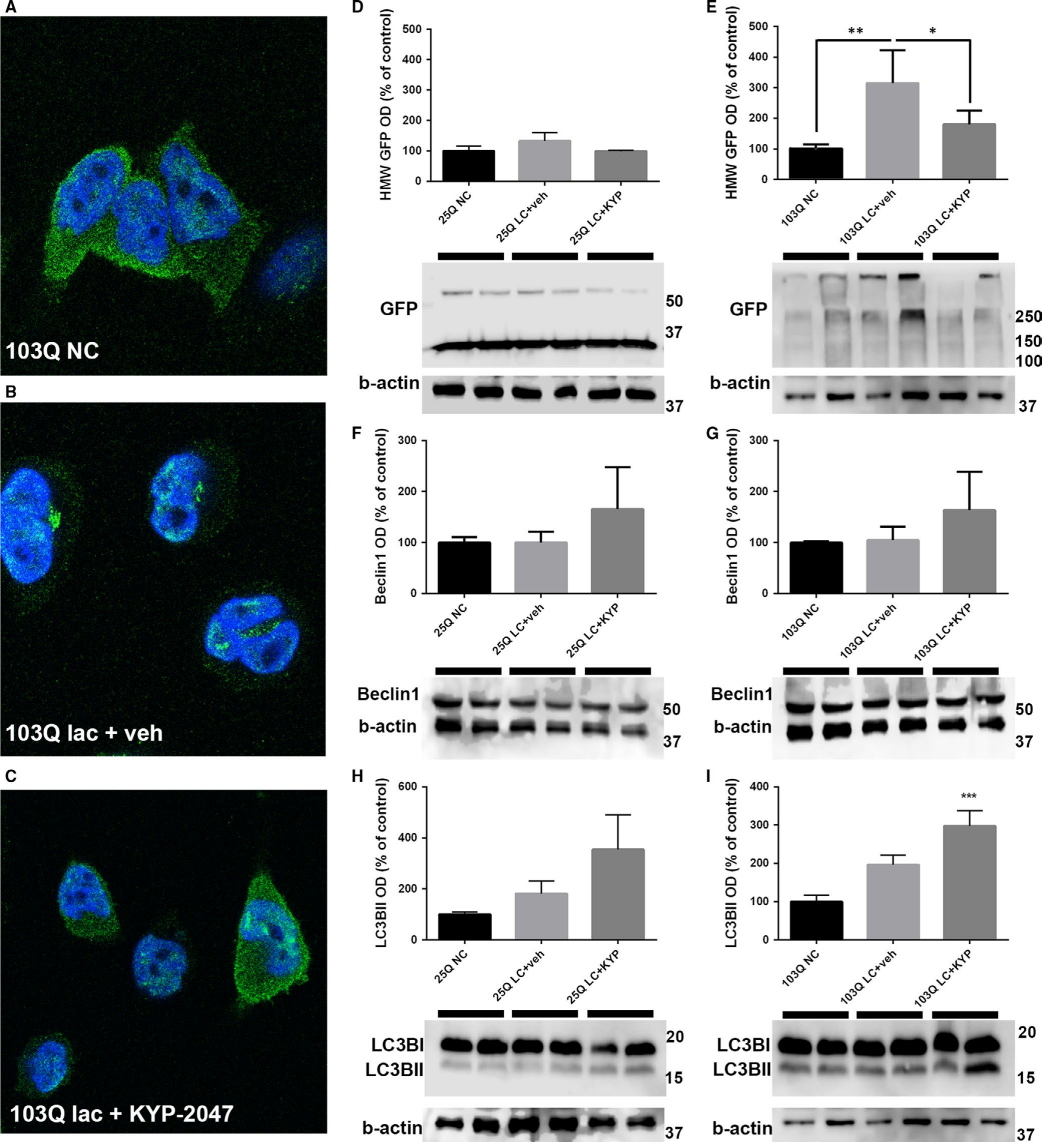
PREP inhibition reduces 103Q aggregation in cells. 103Q cells showed clear aggregates instead of diffuse soluble staining after lactacystin exposure (A‐B), and simultaneous incubation with 1 µmol/L KYP‐2047 reduces cytosolic aggregates (C). Further analysis by Western blot showed significant decrease in insoluble GFP levels (HMW GFP) in 103Q cells while in 25Q cells showed no significant aggregation (D‐E). Beclin1 levels were not significantly increased by KYP‐2047 (F‐G) but LC3BII was significantly elevated in 103Q cells compared to control (I). *P < .05; **P < .01; ***P < .001, 1‐way ANOVA with Tukey post‐test (n of parallel samples = 2; n of individual experiments = 3)

103Q cells were immunostained to detect polyQ aggregates in control and lactacystin‐treated cells. ICC showed that untreated 103Q‐expressing cells had a diffuse staining of mHtt, while addition of 10 µmol/L lactacystin for 48 hours induced the formation of GFP‐positive aggregates that were visible also in nuclei (Figure 2A‐B). Simultaneous incubation of cells with 1 µmol/L KYP‐2047 reduced the cytosolic aggregates (Figure 2C) but did not have an impact on the nuclear aggregates.

Next, we performed immunoblots on cell fractions obtained from 25Q‐ and 103Q‐expressing cells. For this, cells were fractioned into soluble and SDS‐soluble/insoluble fractions as described in Methods. There was a significant increase in high‐molecular‐weight (HMW) mHtt particles, representing insoluble aggregates, in the 103Q expression but not in the 25Q‐expressing cells (Figure 2D). In contrast, the amount of soluble mHtt levels remained largely unchanged (Figure S1).

We have earlier shown that inhibition of PREP can increase Beclin1‐mediated autophagy.^5^ Therefore, we measured the levels of Beclin1 and LC3BII in the soluble fractions of 25Q‐ and 103Q‐expressing cells. Data showed that the conversion of LC3 into active LC3BII in autophagy was significantly increased by KYP‐2047 in lactacystin‐treated 103Q cells (Figure 2I). However, Beclin1 was not significantly changed although KYP‐2047 increased it after lactacystin incubation (Figure 2G). Longer polyQ tracts are reported to increase the degradation of Beclin1^13^ but this was not seen in our study. However, if KYP‐2047 can increase the levels of Beclin1 (as shown in Ref.^5^), this could be beneficial in HD. In 25Q cells, there were no significant alterations in LC3BII or in Beclin1 levels. Additionally, we studied the levels of p62 in soluble fraction but no changes were observed (Figure S1). Together, this suggests that lactacystin induced mainly the aggregation of insoluble mHtt in 103Q‐expressing cells and this was counteracted using KYP‐2047.

In conclusion, our data show that PREP inhibition is able to attenuate the toxicity of 103Q‐mHtt protein by enhancing the autophagy‐mediated clearance of insoluble cytosolic polyQ aggregates as we have shown with aggregated aSyn.^6^ It should be remembered that pharmacological activation of autophagy may under certain conditions cause apoptosis rendering them less attractive for clinical use.^4^ However, the concept of PREP inhibition has been considered safe also in clinical trials^14^ and we propose that targeting PREP and its inhibition can be considered as an attractive possibility for lowering the burden of mHtt and cell toxicity in HD. However, further studies with PREP inhibitors on animal models of HD are needed.

## CONFLICT OF INTEREST

The authors confirm that there are no conflicts of interest.

## AUTHOR CONTRIBUTIONS

DL and TTM planned the study, SN and TTM performed the studies and analysed the data, and SN, DL and TTM wrote the manuscript.

## Data Availability

The data that support the findings of this study are openly available in Figshare at http://doi.org/10.6084/m9.figshare.9741506
