# Supplementary material for: Prolyl oligopeptidase inhibition reduces PolyQ aggregation and improves cell viability in cellular model of Huntington’s disease
Source: J Cell Mol Med. 2019 Sep 29;23(12):8511–5. doi: 10.1111/jcmm.14675 (PMC6850970; doi:10.1111/jcmm.14675)
Supplement: Supplementary file 1 [file JCMM-23-8511-s001.pdf]

Supplementary material for the manuscript "**Prolyl oligopeptidase inhibition reduces PolyQ aggregation and improves cell viability in cellular model of Huntingtin's disease"** by Susanna Norrbacka, Dan Lindholm and Timo T. Myöhänen.

Materials and methods

2.1 Reagents

The PREP inhibitor, KYP-2047 (4-phenylbutanoyl-l-prolyl-2(S)-cyanopyrrolidine), was synthesized in the School of Pharmacy, University of Eastern Finland.[1] KYP-2047 was dissolved as 100 mM in DMSO, and diluted further for the experiments. Lactacystin was purchased from A.G. Scientific (#L-1147; San Diego, CA, USA), dissolved in PBS and used for the experiments as indicated in the Figures.

2.2 Cell cultures

HeLa cells expressing 103Q and 25Q mHtt constructs were cultured in DMEM (Elatus media kitchen, Institute of Biotechnology, University of Helsinki) supplemented with 10% fetal bovine serum (FBS; #16000-044, ThermoFisher Scientific), 1% penicillin-streptomycin solution (P/S; #15140122, ThermoFisher Scientific). Cells expressing Htt with 25Q repeat were used as controls. Cells were kept at 37 °C in 5% CO2 and used for the experiments.

2.3 Cell viability assay

Cells (50,000 cell/well) in 96-well plate were incubated for 48 h with different concentrations of lactacystin (10 nM, 100 nM, 1 µM and 10 µM) in the absence or presence of either 1 µM KYP-2047 or 1 µM DMSO as control. Viability was determined using the Lactate dehydrogenase (LDH) or the 3-(4,5-dimethylthiazol-2-yl)-2,5-diphenyltetrazolium bromide (MTT) assay.

2.4 Western blot

103Q, 25Q and wild-type HeLa cells (400,000 cells/well) in 6-well-plates were incubated with 10 µM lactaystin (+/- 1 µM KYP-2047 or 1 µM DMSO (vehicle)) for 48h to induce Htt aggregation. Cells were lysed using lysis buffer (50 mM Tris-HCl, 100 mM NaCl, 3 mM EGTA and 0.5% Triton X-100) containing HALT phosphatase inhibitor (#87786, ThermoFisher Scientific) and protease inhibitor (#78430, ThermoFisher Scientific). Samples were centrifugated at 13,000 g for 10 min (+4 C), the supernatant removed (soluble fraction) and the remaining pellet was dissolved to SDS-containing 2X Laemmli buffer (insoluble fraction). Standard SDS-PAGE techniques were used and ~30 µg of sample was loaded to 4-20% Mini-Protean TGX gels (#4561094, Bio-Rad) and transferred onto nitrocellulose membranes (#1704159, Bio-Rad). Loading of insoluble fraction was based on soluble fraction protein measurement by BCA. Membranes were incubated at +4 °C overnight in 5% skim milk Tris-buffered saline with 0.05% Tween-20 (TBS-T) with primary antibodies. Following primary antibodies were used: GFP (1:1000; MAB3580; Sigma-Aldrich), Beclin1 (1:1000; ab62557; AbCam, Cambridge, UK), LC3B (1:1000; L-7543; Sigma-Aldrich), p62 (1:5000; ab56416, AbCam) or b-actin (loading control; 1:2500; ab6227, AbCam). After this, the membranes were washed and secondary antibody (1:2000 in 5% skim milk TBS-T) was added for 1h , followed by incubation for 2 h using goat-anti rabbit HRP (LC3B; Beclin1; #31430, ThermoFisher Scientific) or goat anti-mouse HRP (GFP, p62; #31463, ThermoFisher Scientific). Images were captured using the C-Digit imaging system (Licor, Lincoln, NE), and the optical densities (OD) of the bands were measured by ImageJ (8-bit grayscale image; version 1.48; National Institute of Health, Bethesda, MD). β-actin (ab8227, AbCam) was used as a control, and experiments done at least three times.

2.5 Immunocytochemistry (ICC)

25Q- and 103Q- expressing cells (100,000 cells/well) were plated onto glass coverslips in a 12-well plate, allowed to attach overnight, and subsequently treated for 48h with 10 µM lactacystin +/- 1 µM

KYP-2047 or 1 µM DMSO (vehicle). Cells were fixed with 4% paraformaldehyde for 20 min at RT, blocking solution, 10% normal goat serum (S-1000, Vector Laboratories, Peterborough, United Kingdom) was added for 30 min, followed by incubation with anti-GFP (1:1000 in 1% normal goat serum) overnight at +4 °C. After washing, anti-mouse AlexaFluor488 (dilution 1:400 in 1% normal goat serum; ab150113, Abcam) secondary antibodies were added for 1 h. Cells were washed and mounted with Vectashield containing DAPI to stain nuclei (H-1200, Vector Laboratories). Imaging was performed using Leica TCS SP5 confocal microscope (Leica Microsystems, Wetzlar, Germany), and minor modifications to brightness and contrast were made.

Supplementary figures

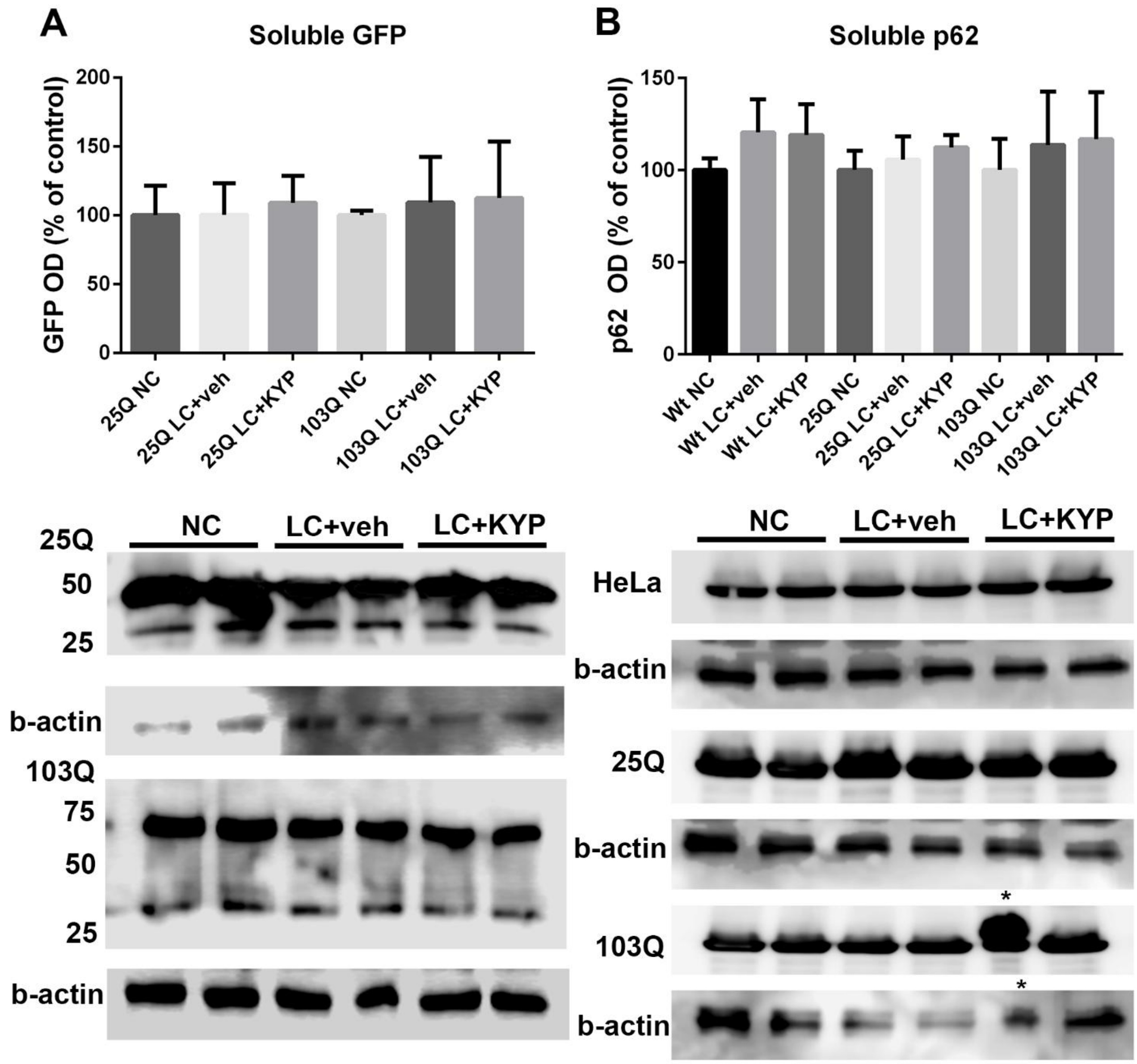

Supplementary Figure S1. 48h lactacystin incubation did not significantly alter the levels of soluble mHtt assayed by GFP immunoblotting (A) or the levels of soluble p62 (B). *, unsuccessful band, not included in the analysis.

References

1. **Jarho EM, et al.** A cyclopent-2-enecarbonyl group mimics proline at the P2 position of prolyl oligopeptidase inhibitors. *J Med Chem*. 2004; 47: 5605-5607.
